# Intracranial Mass Lesion in a Patient Being Followed up for Amblyopia

**DOI:** 10.4274/tjo.galenos.2020.36360

**Published:** 2020-10-30

**Authors:** Ali Mert Koçer, Bayazıt İlhan, Anıl Güngör

**Affiliations:** 1 Ulucanlar Ophthalmology Trainig and Research Hospital, Clinic of Ophthalmology, Ankara, Turkey

**Keywords:** Amblyopia, craniopharyngioma, hemianopsia

## Abstract

A 12-year-old boy being followed up for amblyopia presented to our hospital with visual disturbance in the left eye. The patient’s best corrected visual acuity on Snellen chart was 1.0 in the right eye and 0.3 in the left eye. Increased horizontal cup-to-disc ratio was detected on dilated fundus examination. Retinal nerve fiber layer measurement showed diffuse nerve fiber loss and visual field test showed bitemporal hemianopsia. Magnetic resonance imaging revealed a lesion that filled and widened the sella and suprasellar cistern and compressed the optic chiasm. The patient was operated with transcranial approach. The pathologic examination revealed craniopharyngioma.

## Introduction

Amblyopia is poor best corrected visual acuity (BCVA) in one or both eyes due to low vision or abnormal binocular interaction without any detectable structural defect in the eye or visual pathways. Amblyopic vision loss can be corrected if treated at an early age. The most important factors in the development of amblyopia are the severity, timing of onset, and duration of visual impairment. Blurred vision is much more likely to develop into amblyopia in young children. Although variable, the risk of amblyopia is generally higher in the first 2-3 years of life, and the risk is reported to continue until the age of 12.

Anisometropia and strabismus are among the most common causes of amblyopia.^1^ Blurred vision caused by uncorrected refractive error appears to be the main factor that prevents the development of central vision in anisometropic amblyopia. Anisometropia is generally defined as a difference in spherical/cylindrical refractive errors of 1.5-2 diopters (D) or more and is more common in hyperopic eyes than in myopia.^2^

Craniopharyngioma is a benign tumor that develops from the remnant of Rathke’s pouch and is located in the sellar/parasellar region.^3^ It shows a bimodal age distribution, with patients usually diagnosed between the ages of 5 and 14 or after the age of 50.^4^ Although these tumors are slow-growing and benign, they can cause serious symptoms due to their proximity to important anatomical structures such as the pituitary gland, hypothalamus, and optic chiasm. Clinical manifestations may include endocrine disorders such as hypothyroidism, impotence, and amenorrhea; visual symptoms such as optic atrophy, visual field defect, and decreased vision; and symptoms related to increased intracranial pressure, such as headache, nausea, and vomiting.^5,6^ The most frequent ocular signs are bitemporal hemianopsia and optic atrophy. Diagnosis of craniopharyngioma is based on neurological, visual, and endocrine symptoms and the appearance of a calcific solid/cystic lesion on radiologic imaging. The diagnosis is confirmed by pathologic examination.

In this article, we present a patient under follow-up for amblyopia who was diagnosed as having craniopharyngioma after further examination in our clinic.

## Case Report

**Figure 1:**
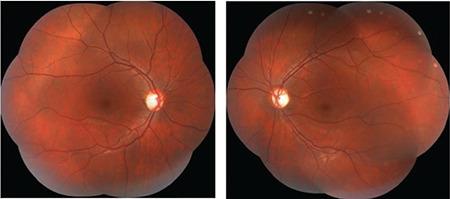
Bilateral fundus photographs show a normal macula and increased cupto- disc ratio in the optic disc

**Figure 2:**
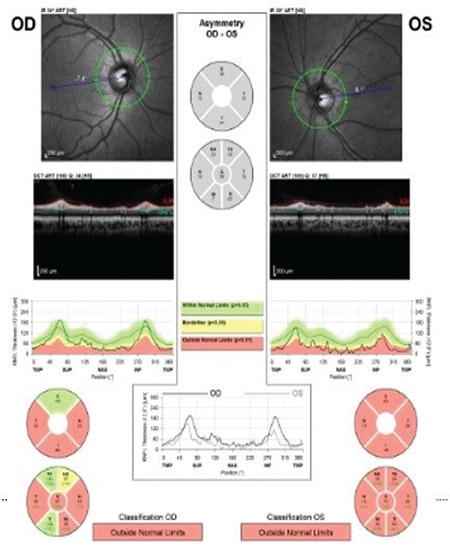
Retinal nerve fiber layer thickness measurement demonstrated diffuse retinal nerve fiber loss in both eyes

**Figure 3:**
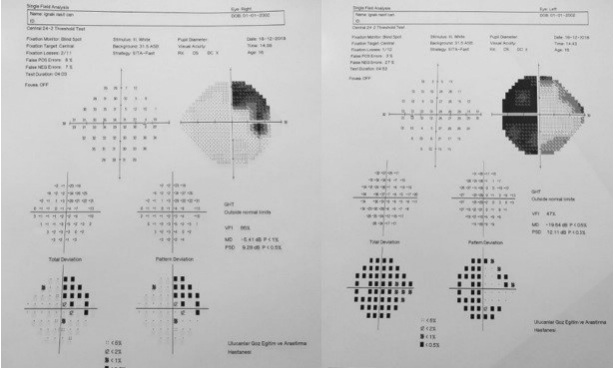
The 24-2 visual field test revealed visual field defect that was more prominent in the left eye and bitemporal hemianopsia

**Figure 4:**
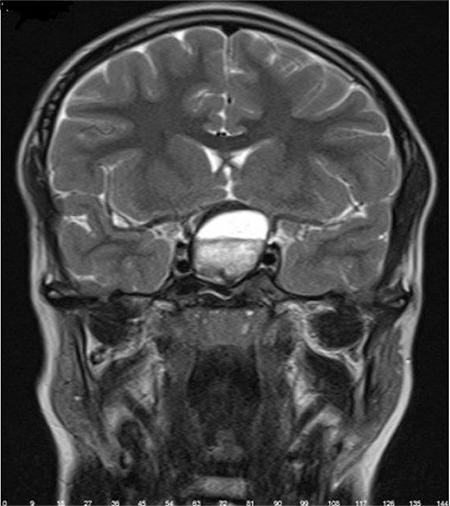
In the cranial magnetic resonance imaging coronal slice, a sellar/ suprasellar intracranial mass was observed

A 12-year-old boy with no disease, history of trauma, or systemic symptoms presented to our hospital with low vision in his left eye. It was learned that the patient was under follow-up at another center for left amblyopia and had been treated with right eye closure for a period of time. His family history included no consanguineous marriage or illnesses that cause vision impairment. Written informed consent was obtained from the patient for the examination and tests. Autorefractometer measurements showed a spherical refraction error of -0.25 D in the right eye and -1.5 D in the left eye. His Snellen BCVA was 1.0 in the right eye and 0.3 in the left eye. Intraocular pressure was measured as 15 mmHg in both eyes. No afferent pupillary defect was detected. Color vision in both eyes was evaluated as normal using the Ishihara color vision test. Anterior segment examination findings were normal. On dilated fundus examination, both posterior poles appeared normal, whereas bilateral optic disc pallor and increased horizontal cup-to-disc (C/D) ratio were observed (Figure 1). Retinal nerve fiber layer (RNFL) thickness measurement (Spectralis, Heidelberg Engineering, Heidelberg, Germany) revealed nerve fiber loss around both optic discs (Figure 2). The macula appeared normal in both eyes on optical coherence tomography (Spectralis, Heidelberg Engineering, Heidelberg, Germany). Bitemporal hemianopsia that was more prominent on the left side was observed in 24-2 visual field test (Humphrey Field Analyzer; Carl Zeiss Meditec, Dublin, CA) (Figure 3). Suspecting the patient may have an intracranial lesion compressing the optic chiasm, cranial and pituitary magnetic resonance imaging (MRI) was requested. MRI demonstrated a nonenhancing lesion 24 x 23 x 32 mm in size filling and widening the sella and suprasellar cistern and compressing the optic chiasm. On T1- and T2-weighted images, the lower half of the mass was heterogeneous and the upper half was hypointense (Figure 4). Craniopharyngioma and hemorrhagic complicated adenoma were considered in the radiologic differential diagnosis. The patient underwent surgery via transcranial approach in the neurosurgery department. On pathologic examination of the specimen, diffuse hyalinization, calcification, xanthogranulomatous debris, and several foci of keratin adjacent to the adenohypophysis were observed and the lesion was diagnosed as craniopharyngioma. The patient was evaluated with visual field test until postoperative month 6 and no changes were detected in visual field or BCVA.

## Discussion

Amblyopia is unilateral or bilateral low best corrected visual acuity (BCVA) caused by poor vision or abnormal binocular interaction in the absence of a structural defect in the eye or visual pathways.^7^ Excluding organic pathologies that can lead to optic neuropathy, such as intracranial masses, before making a diagnosis of amblyopia is critical in the follow-up and treatment approach. In the pediatric population, the most common intracranial masses include astrocytoma, ependymoma, and medullablastoma. In addition, considering that in many studies the most common symptom of craniopharyngioma was blurred vision and subsequent decrease in visual acuity, craniopharyngioma seems to be a pathology that should be considered in the differential diagnosis of intracranial masses.^8,9^ Especially in patients with vision loss accompanied by symptoms such as headache, nausea/vomiting, growth retardation, delay in the development of secondary sex characteristics, polydipsia, and polyuria, it is important to perform a systemic examination and to consider an organic pathology as the cause of low vision. If there are no accompanying systemic symptoms, as in our patient, a careful fundus examination and the use of auxiliary imaging methods when necessary can provide important information about the cause of low vision. Although craniopharyngioma is a benign tumor, diagnosis and treatment are important due to its close proximity to important anatomical structures. These tumors are most commonly located in the sellar/parasellar region.^10^ The tumor typically places pressure on the optic chiasm from the anterior and posterior, generally resulting in bitemporal hemianopsia visual field defect. In some cases, homonymous hemianopsia can also be seen.^11^ Pituitary tumor, suprasellar aneurysm, third ventricular glioma, and tuberculum sellae meningioma are among the pathologies that cause bitemporal hemianopsia visual field defect and should be included in the differential diagnosis of craniopharyngioma.

The surgical treatment of craniopharyngioma involves resection of the tumor via a transcranial or transsphenoidal approach while preserving the optic and hypothalamic structures. Total resection or subtotal resection with or without radiotherapy is performed as appropriate according to parameters such as tumor size, growth pattern, and hypothalamus involvement.^12,13^ Initial symptoms are visual in more than half of patients, and about 41-48% of patients may have some amount of visual gain after surgery.^14^ Poor prognostic factors in postoperative visual gain are severe vision loss and prechiasmatic tumor location.^15^ Although the transsphenoidal surgical approach is associated with better visual prognosis, it is only effective in intrasellar tumors. In addition, suprasellar positioning is seen in a larger proportion of craniopharyngioma patients.

Undergoing surgery via transcranial approach and having very low preoperative visual acuity seem to be poor prognostic factors in our case. Our patient showed no changes in visual field or BCVA in postoperative evaluations, demonstrating the importance of early diagnosis of intracranial masses causing optic neuropathy in terms of postoperative visual recovery. We believe that early diagnosis may have been possible in our patient with the detection of temporal optic disc pallor and increased C/D ratio, but the diagnosis was delayed because these changes were not detected in the early period. Furthermore, it should not be forgotten that patients diagnosed with intracranial mass may exhibit some amount of anisometropic amblyopia, as in the present case.

In conclusion, because diagnosis occurs later in children, visual morbidity is greater, and visual acuity generally remains low after treatment at more advanced stages, early diagnosis of tumors is important to minimize visual sequelae. The most important steps for early diagnosis are a detailed history, careful fundus examination, and visual field testing when necessary. In children with suspicious examination findings and inadequate cooperation, we recommend using additional tests before a diagnosis of amblyopia is made.
